# Determination of Pharmaceutical Residues by UPLC-MS/MS Method: Validation and Application on Surface Water and Hospital Wastewater

**DOI:** 10.1155/2021/6628285

**Published:** 2021-01-08

**Authors:** Bui Van Hoi, Cam-Tu Vu, Lan-Anh Phung-Thi, Thao Thi Nguyen, Phuong Thanh Nguyen, Huong Mai, Phuong-Thu Le, Thanh-Hien Nguyen, Dao Thanh Duong, Hue Nguyen Thi, Dung Le-Van, Dinh Binh Chu

**Affiliations:** ^1^Department of Water–Environment-Oceanography, University of Science and Technology of Hanoi (USTH), Vietnam Academy of Science and Technology, 18 Hoang Quoc Viet, Cau Giay, Hanoi 100000, Vietnam; ^2^School of Environmental Science and Technology, Hanoi University of Science and Technology, No. 1 Dai Co Viet, Hanoi 100000, Vietnam; ^3^FPT University, Hoa Lac High Tech Park, Km 29 Thang Long Boulevard, Thach That, Hanoi 100000, Vietnam; ^4^Institute of Environmental Technology, Vietnam Academy of Science and Technology, 18 Hoang Quoc Viet, Cau Giay, Hanoi 100000, Vietnam; ^5^Department of Chemistry, Vietnam Military Medical University, 160 Phung Hung, Ha Dong, Hanoi 100000, Vietnam; ^6^School of Chemical Engineering, Hanoi University of Science and Technology, No. 1 Dai Co Viet, Hanoi 100000, Vietnam

## Abstract

In this study, an analytical method for the simultaneous determination of 7 major pharmaceutical residues in Vietnam, namely, carbamazepine, ciprofloxacin, ofloxacin, ketoprofen, paracetamol, sulfamethoxazole, and trimethoprim, in surface water and hospital wastewater has been developed. The method includes enrichment and clean-up steps by solid phase extraction using mix-mode cation exchange, followed by identification and quantification using an ultrahigh-performance liquid chromatography and tandem mass spectrometry and employing electrospray ionization (UPLC-ESI-MS/MS). Seven target compounds were separated on the reversed phase column and detected in multiple reaction monitoring (MRM) mode within 6 minutes. The present study also optimized the operating parameters of the mass spectrometer to achieve the highest analytical signals for all target compounds. All characteristic parameters of the analytical method were investigated, including linearity range, limit of detection, limit of quantification, precision, and accuracy. The important parameter in UPLC-ESI-MS/MS, matrix effect, was assessed and implemented via preextraction and postextraction spiking experiments. The overall recoveries of all target compounds were in the ranges from 55% to 109% and 56 % to 115% for surface water and hospital wastewater, respectively. Detection limits for surface water and hospital wastewater were 0.005–0.015 *µ*g L^−1^ and 0.014–0.123 *µ*g L^−1^, respectively. The sensitivity of the developed method was allowed for determination of target compounds at trace level in environmental water samples. The in-house validation of the developed method was performed by spiking experiment in both the surface water and hospital wastewater matrix. The method was then applied to analyze several surface water and hospital wastewater samples taken from West Lake and some hospitals in Vietnam, where the level of these pharmaceutical product residues was still missed. Sulfamethoxazole was present at a high detection frequency in both surface water (33% of analyzed samples) and hospital wastewater (81% of analyzed samples) samples.

## 1. Introduction

Pharmaceutical residues are nowadays considered as an emerging group of organic pollutants in aqueous environment [1]. These compounds can be accumulated in aquatic biota [2]. Then, these compounds can reach humans via the food chain. However, analysis of pharmaceutical residues in environmental samples, especially water samples, is still challenging because these compounds are of low concentrations in real samples and have a high polarity and fast interconversion, and the sample matrix is of a high complexity [3]. Analytical methods have already been developed and published to quantify these pollutants in the aqueous environment, for example, gas chromatography combined with mass spectrometry after on-fiber derivatization (SMPE-GC-MS) [4]; combination of liquid chromatography and tandem mass spectrometry (LC-MS/MS) [5, 6]; two-dimensional liquid chromatography (LC x LC-MS) [7]. Among those methods, LC-MS or LC-MS/MS method was the most popular for quantification of such compounds in water samples [8, 9]. In addition, the liquid chromatography combined with high resolution mass spectrometry (time of flight mass spectrometry and Orbitrap mass spectrometry) based method has been also developed for both targeted and nontargeted analysis in water or in sediment samples [10–12]. However, liquid chromatography in combination with tandem mass spectrometry (LC-MS/MS) was used in most laboratories because of its sensitivity, selectivity, and robustness [13, 14]. In addition, because of the low concentrations in water and environmental samples, several procedures for sample preparation have been investigated for enriching the concentration of target compounds in samples such as liquid extraction, solid phase extraction, accelerated solvent extraction, and microwave-assisted extraction [15–18]. Because of its advantages, solid phase extraction has been the most popular for enrichment and clean-up in sample preparation for LC-MS/MS to analyze the pharmaceutical residues in environmental samples, especially liquid samples [19–21].

In this study, seven major pharmaceutical residues in surface water and hospital wastewater, including carbamazepine, ciprofloxacin, ofloxacin, ketoprofen, paracetamol, sulfamethoxazole, and trimethoprim, were analyzed using ultrahigh performance liquid chromatography (UPLC) combined with positive ionization tandem mass spectrometry (MS/MS) in multiple monitoring reaction mode. These compounds were commonly reported as the most popular antibiotics in Vietnam in recent publications [22–24]. Water sample was cleaned up with multimode solid phase extraction (cation exchange and reversed phase mode in the same solid phase extraction cartridge). An important parameter in electrospray ionization tandem mass spectrometry, ionization suppression/enhancement, was fully investigated by preextraction and postextraction spiking experiments. The developed method was in-house validated by standard addition experiments. This method was then applied for screening of pharmaceutical residues in surface water and hospital wastewater samples, which were collected from some areas in Vietnam.

## 2. Materials and Methods

### 2.1. Chemicals

Carbamazepine (CARBA), ciprofloxacin (CIPRO), ofloxacin (OFLO), sulfamethoxazole (SULFA), trimethoprim (TRIM), ketoprofen (KETO), paracetamol (PARA), and organic solvents, acetonitrile (MeCN) and methanol (MeOH), were purchased from Sigma-Aldrich (Singapore, LC MS grade). Internal standard, sulfamethoxazole-^13^C_6_ (SULFA-13C6), ofloxacin-D3 (OFLO-D3), and paracetamol-D4 (PARA-D4) were supplied by Toronto Research Chemicals (TRC, Toronto, Canada). Formic acid (FA) 100% (Optima MS grade) and ammonium hydroxide solution 25% (reagent grade) were provided from Merck (Germany). Oasis mix-mode cation exchange (MCX, 3 cc, 60 mg) and hydrophilic lipophilic balance (HLB, 6 cc, 200 mg) solid phase extraction cartridges were purchased from Waters (USA), while strong cation exchange cartridge (Supelco, SCX, 6 cc, 200 mg) was purchased from Sigma Aldrich (Singapore). Ultrapure water (18.3 MΩ cm) from Smart2pure 12 UV water purify system (Thermo, England) was used throughout this study.

Individual stock standard solution was prepared by dissolving an appropriate amount of analyte in MeCN at 10 mg L^−1^. In case of CIPRO and OFLO, 100 *µ*L of 10 M NaOH was added for complete dissolution. Then, working standard solution was prepared by dilution in MeCN for optimization of operating parameters of the mass spectrometer via direct infusion analysis. All stock solutions were stocked in amber glass tube at −20°C. The working standard solution was daily prepared by mixing of single stock solutions in mixture of acetonitrile and water containing 0.5% formic acid (mobile phase). Mobile phase was daily prepared by adding a volume of concentrated formic acid in MeCN or ultrapure water. Mobile phase was filtered using the syringe filter with a pore size of 0.2 *µ*m. The mobile phases were degassed in an ultrasonic water bath in order to remove dissolved gas.

### 2.2. Sample Preparation

#### 2.2.1. Surface Water

**Figure 1:**
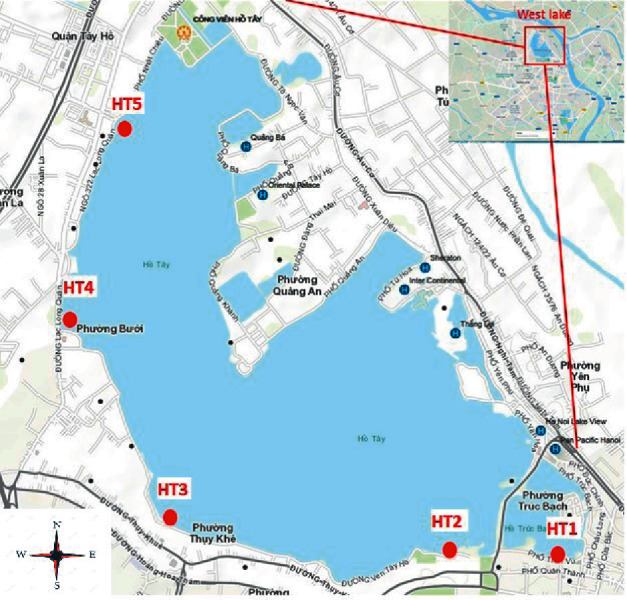
Surface water sampling points at the West Lake.

The study area is West Lake, the biggest lake in Hanoi, which covers 5.3 km^2^ watershed. This watershed mainly receives water from Red River as well as the domestic and hospital wastewater located at the surrounding area. All samples were collected and kept in plastic bottles (1 L) prerinsed with ultrapure water in the laboratory and rinsed with sample on the field. Four sample points (as shown in Figure 1) were chosen to collect water samples in this lake, and the samples were kept at 4°C using glaciers and ice bags and brought back directly to the laboratory within the sampling day. In parallel, one more sample was collected from Truc Bach Lake, which is close to West Lake. The sampling site is 30 m from the discharge point of wastewater treatment plant Truc Bach, which is treating 2500 m^3^ day^−1^ of domestic wastewater.

#### 2.2.2. Hospital Wastewater

Hospital wastewater samples (*n* = 11), including five influents (IN) and six effluents (EF), were collected from six different hospital treatment stations in North Vietnam, Lang Son (LS_EF) and Hanoi (ND_IN, ND_EF, RHM_EF), and South Vietnam, Can Tho (CT_IN) and Ho Chi Minh City (NHD1_IN, NHD2_IN, NHD3_IN, NHD_EF, CR1_EF, CR2_EF), according to US EPA 1694 guideline with some modifications. All samples were collected and kept in plastic bottles (1 L) prerinsed with ultrapure water in the laboratory and rinsed with sample on the sampling site. The samples were kept at 4°C using glaciers and ice bag and brought directly to the laboratory on the day of sampled collection or kept at −20°C and brought back to the laboratory on another day. In laboratory, samples were filtered by using microglass fiber filters (GF/F Whatman, *ϕ* ≤ 0.7 *µ*m) helped by vacuum filtration unit to eliminate suspended matters. The prefilter (GF/A Whatman, *ϕ* ≥ 1.6 *µ*m) was used if the samples have greatly been charged by suspended matters. All glass microfiber filters have been treated by baking at 450°C for 4 h in order to eliminate all organic contaminants. The filtered samples were stored in −20°C and then either extracted and analyzed within 48 h after collection or kept at −80°C until analysis.

#### 2.2.3. Sample Preparation for LC-MS/MS Analysis

Filtered surface water and wastewater samples were thawed and placed at room temperature before spiking the internal standard. The isotopic labelled internal standard (final concentration in each internal standard was 50 ng mL^−1^) was spiked into samples at ambient temperature for one hour before treatment. The Waters Oasis MCX (mixed mode cation exchange and reversed phase materials) solid phase extraction cartridges were conditioned using 3 mL of MeOH, followed by 2 × 3 mL of acidified water (pH 3.0 adjusted with 2 M FA). A 200 mL of surface water or hospital wastewater was adjusted to pH 3 with 2 M FA before loading on SPE cartridge by applied vacuum. The flow rate of loading sample on the SPE cartridge was 12–15 mL min^−1^. The MCX SPE cartridges were then washed with 3 mL of water (pH 3.0) to remove interferences and dried for 30 minutes under vacuum. The elution step was performed with 5 × 1 mL of mixture of MeOH/2M NH_4_OH (90/10; v/v). The extract was evaporated under gentle nitrogen stream until dryness and then dissolved to 1 mL of H_2_O/MeCN (95/5; v/v). Finally, the solutions were filtered using the syringe filter with 0.2 *µ*m pore size and injected into UPLC-ESI- MS/MS system at the optimal operating conditions. Other solid phase extraction materials such as HLB (Waters, USA) and SCX (Sigma, Singapore) were also tested to investigate enrichment of pharmaceutical residues in both surface water and wastewater samples.

### 2.3. LC-MS/MS Measurement

#### 2.3.1. LC-MS/MS Conditions

An ultrahigh performance liquid chromatography (ACQUITY UPLC, H-class, Waters, USA) in combination with a tandem mass spectrometer (Xevo TQD, Waters, USA) was used for the analysis. Target compounds were separated on a reversed phase chromatography column (BEH C18 column, 130 Å, 1.7 *µ*m, 2.1 mm i.d. x 50 mm) accompanied with a guard column (3.5 µm, 2.1 mm i.d. x 10 mm; Waters, USA). The analyte was eluted using a gradient of mobile phase containing 0.5% FA in water (mobile phase A) and 0.5% FA in MeCN (mobile phase B). Gradient of mobile phase was linearly scheduled as follows, 100% A for 0-1 min and a linear increase to 70% B from 1–3 min, and then, it was kept for 1 min and decreased to initial mobile phase condition for reequilibrium column. The total chromatographic separation time was 6 min. A 10 *µ*L of sample or standard solution was injected on the analytical column via an autosampler. The flow rate of mobile phase was constantly kept at 300 *µ*L min^−1^. The temperatures of analytical column and sample tray were kept at 30°C and 10°C, respectively.

The target compounds were detected by multiple reaction monitoring (MRM) mode in positive electrospray ionization (+ESI). The optimum conditioning of the ionization source was set up as follows: desolvation temperature at 350°C, source temperature at 150°C, cone gas flow at 645 L h^−1^, desolvation gas flow at 10 L h^−1^, and capillary voltage at 3, 0 kV. The optimization of transitions in MS/MS mode was performed by direct infusion using single standard solution in mobile phase via IntelliStart function in MassLynx version 4.1 (Waters, USA). The operating experiments for all target compounds are listed in Table 1.

#### 2.3.2. Matrix Effect in UPLC-MS/MS

In the analytical method, the matrix of sample is the most important factor that affects the reproducibility. Several designs of experiments were proposed to assess the matrix effect, for instance, postextraction spiking experiments, matrix match calibration curves, and isotopic labelled internal standard spiking experiments [25, 26]. The standards were spiked before and after extraction during sample preparation procedure. Due to the difference in sample matrix, the spiking experiments were conducted on both surface water and hospital wastewater samples. In this study, the postextraction spiking experiments were applied to investigate the matrix effect (ME) in terms of signal suppression/enhancement. Particularly, ME was compared using the signals of analytes in samples spiked after extraction (in sample matrix solution) and analytes in the “neat” solvent. The matrix effect, extraction efficiency, and overall recovery were calculated as Vu-Duc et al. proposed [27]. Briefly, the ME, ionization enhancement, or suppression in mass spectrometry was assessed via postextraction spiking experiments. The analytical signal of the analyte in the sample matrix was used for comparison with the analytical signal of that in “neat” solvent.

### 2.4. In-House Method Validation

The validation of method was investigated using different parameters, which have been proposed by Peter et al., including linearity range, accuracy and precision, extraction recovery, method detection limits (MDLs), method quantification limits (MQLs), quality control (QC), and accuracy and precision expressed as relative standard deviation (%RSD) [28]. Linearity range was investigated by setting calibration curves with concentration in range from 5 to 500 *µ*g L^−1^ (with seven calibration levels). The quantification of the target compounds was performed by isotopic labelled internal standards. The precision of method was performed by injecting three QC samples at three levels of concentration (low, medium, and high) relative to calibration range and by interday and intraday. The MDLs and MQLs were determined using the signal-to-noise ratio 3 and 10, respectively, and on the real sample matrix. For recovery testing, the spiking experiments were conducted on both surface water and hospital wastewater samples. The volume of sample taken to analysis was 200 mL for both surface water and hospital wastewater.

### 2.5. Quality Control

For the quality control, 20% of total injections was quality control (QC) sample and blank sample. The QC sample was prepared by dilution of stock standard solution in a mixture of water and acetonitrile (95/5; v/v) at 50 ng mL^−1^ and four internal standards at the same concentration level. The blank sample contained only internal standards at 50 ng mL−1 in a mixture of acetonitrile and water (95/5; v/v). The stability of the analytical signal was monitored by both QC samples and tuning solution. The QCs were then injected by regular intervals as proposed by Want et al. [29]. Briefly, the performance of the mass spectrometry was checked by using tuning solution before one batch injection. One QC sample was injected after 10 injections of the sample including the blank. The carry-over effect during injection of each batch was assessed by the blank sample that was prepared as a mobile phase. The presence of target compounds in real samples was identified by three factors: retention time, two transitions in MRM mode, and relative intensity of two transitions of each compound. Therefore, identification points of all analytes have achieved at least four points accordingly to European Union Guidance for the validation of the analytical method [30]. Data evaluation and calculation were performed using TargetLynx XS in Masslynx version 4.1 (Waters, USA) and Microsoft Excel 2020 (Microsoft, USA).

## 3. Results and Discussion

### 3.1. Optimization of UPLC-MS/MS for Target Compounds

**Figure 2:**
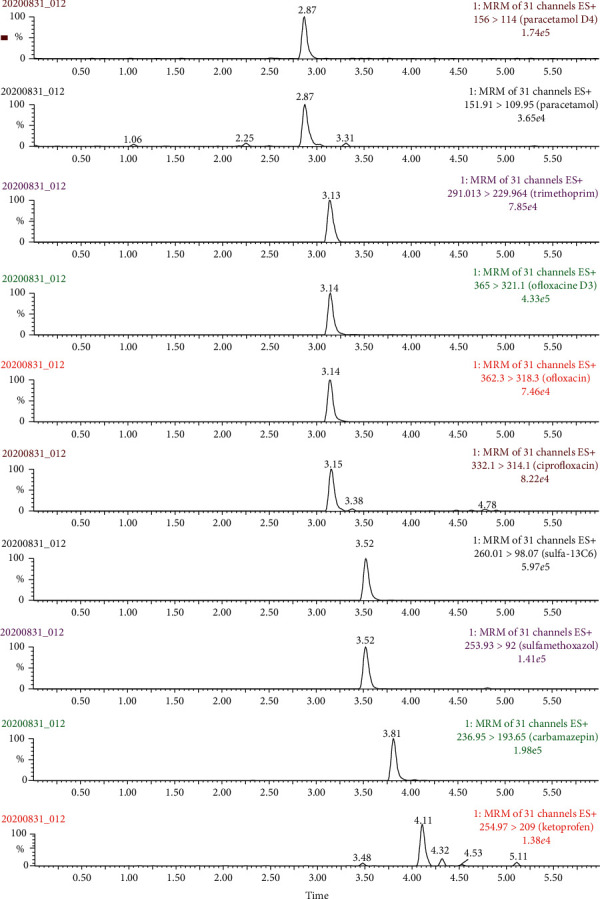
Extracted ion chromatograms of all standard compounds at 100 *µ*g L^−1^ except IS at 50 *µ*g L^−1^.

Different experiments were performed in order to optimize chromatographic separation for all compounds using Waters BEH C18 reversed-phase column. Several mobile phase gradients were evaluated using MeCN and MeOH as the organic phases and ultrapure water as the aqueous phase. FA was added to the mobile phase in order to provide proton for promoting + ESI process. Briefly, the mobile phases used contained H_2_O (phase A) and MeCN (phase B), both containing 0.5% FA (v/v). The flow rate and column oven temperature were both optimized to improve the peak shape and chromatographic resolution efficiency. The optimized conditions were finally set at 300 *µ*L min^−1^ and 30°C for flow rate and column oven temperature, respectively.  Figure 2 shows the extracted ion chromatograms at 100 *µ*g L^−1^ for target compounds and 50 *µ*g L^−1^ for internal standards.

As clearly shown in Figure 2, all target compounds were well retained on the Waters BEH C18 column, and peak shape (asymmetry factor) is in the acceptable range (from 0.8 to 1.2) according to European Pharmacopeia standards (Section 2.2.46 Chromatographic separation techniques).

In addition, operating parameters of the mass spectrometer also were investigated in order to achieve the highest sensitivity for all target compounds. Multiple reaction monitoring (MRM) was used to measure the product ions of the protonated molecular ion for each compound. The transition information of each compound including isotopic labelled internal standard in MRM mode is shown in Table 1. In this mode, two characteristic MRM transitions with highest intensity were selected for each compound in order to achieve the highest sensitivity and selectivity. The relative intensity of two MRM transitions in MS and retention time on chromatographic column was critically used to identify the presence of analytes in samples as suggestion from Angeles and Aga [31].

### 3.2. In-House Method Validation

#### 3.2.1. Linearity Range and Sensitivity

Seven standard solutions (concentration from 5 *µ*g L^−1^ to 500 *µ*g L^−1^ of target analyte and 50 *µ*g L^−1^ for internal standards) were prepared in mobile phase and were injected in triplicate into UPLC- MS/MS. The ratio of peak area is calculated by dividing the peak area of compounds by the peak area of appropriate isotopic labelled internal standard. The mean value of peak area ratio (*y*) was fitted as an unweighted linearity function of concentration (*x*) of compounds. The limit of detection (LOD) and limit of quantification (LOQ) were calculated as three and ten times the signal-to-noise ratio, respectively [32, 33]. Regression equation, correlation coefficient (*R*^2^), and method detection limit are shown in Table 2.

As clearly shown in Table 2, a strong correlation between peak area ratio and concentration (*R*^2^ >0.99) was achieved for all compounds. In addition, the sensitivity of the developed method was compared with recent publications, and there was enough sensitivity for the analysis of pharmaceutical residues in both surface water and hospital wastewater [13, 22, 34].

#### 3.2.2. Stability of Analytical Signal

**Figure 3:**
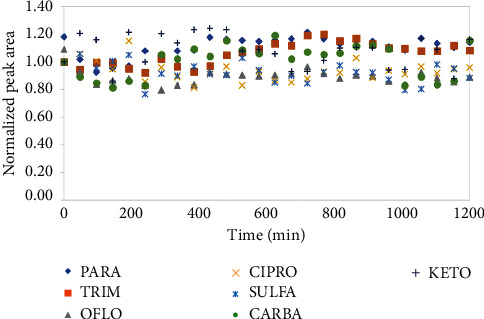
Stability of the analytical signal of all target compounds for 20 hours of injection.

Another critical parameter of the analytical method is the stability of the instrument and standard compounds during injection. To examine that, a mixture of standards at 100 µg L−1 was prepared in the mobile phase and injected to UPLC-MS/MS system. The temperature of standards was kept at 4°C, and they were continuously injected for 20 hours. The normalized peak areas of all target compounds were taken into account as a linearity function of injection time as in Figure 3.

The linearity regression equations fitted by a normal peak area as a function of injection time of all compounds were calculated. The results showed that slopes of all regression equations are not significantly different from “zero” value. It could be concluded that all target compounds were stable at the injection testing conditions [28].

**Figure 4:**
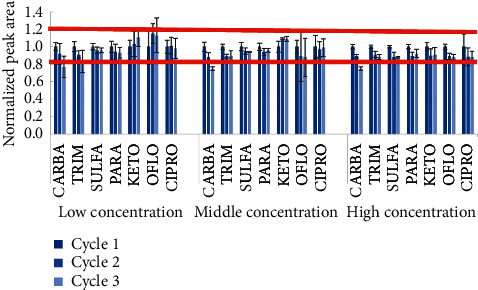
Stability of target compounds in three thawing-freezing cycles at 10 *µ*g L^−1^ (low concentration); 200 *µ*g L^−1^ (middle concentration); 500 *µ*g L^−1^ (high concentration).

In addition, the stability of standards during storage was also investigated. The stability of standards in the mobile phase in three thawing-freezing cycles was performed according to the US-FDA guideline for the validation of an analytical method [35]. The normalized peak areas of all analytes at three concentration levels were taken into account for stability of the standards. The stability of standards during injection time is demonstrated in Figure 4.

As clearly shown in Figure 4, normalized peak areas of all targeted analytes are stable after three thawing-freezing testing cycles. Interestingly, the higher relative standard deviation was observed at a lower concentration for all target compounds. SULFA has given the best stability, while KETO and CIPRO had a low stability during 20 h of injection. However, the normalized peak areas of all target compounds fell in the acceptable range. This indicated that the thawing-freezing cycles did not affect the stability of the target compounds [36, 37].

#### 3.2.3. Optimization of Sample Preparation

**Figure 5:**
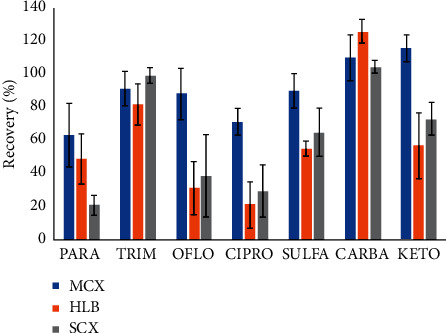
Recoveries of seven pharmaceutical products in surface water on different solid phase extraction materials.

In this study, three different kinds of solid phase extraction materials, hydrophilic-lipophilic balance sorbent (Oasis HLB, Waters, USA), mixed mode cation exchange sorbent (MCX, Waters, USA), and strong cation exchange sorbent (SCX, Sigma, Singapore), were chosen for enrichment and clean-up. All SPE cartridges were treated as recommendation from manufactory. All experiments were conducted in triplicate. Blank sample was prepared by using tap water instead of real water sample. In general, SPE cartridge was conditioned by MeOH (MCX, SCX column) or MeCN (HLB column), followed by acidified water. The acidified sample was loaded on the SPE cartridge at approximately 12–15 mL min^−1^ using vacuum and washed with 5 mL of acidified water (pH 3.0) to remove chemical interferences. The SPE cartridge was then dried under low pressure for 30 minutes. Finally, target compounds on the SPE cartridge were eluted by suitable solvent composition (5 × 1 mL of MeOH/2M NH_3_, 90/10, v/v for MCX and SCX cartridge and 5 × 1 mL of MeCN/H_2_O, 60/40, v/v for HLB cartridge). The eluents were then collected in 15 mL glass tube and dried under gentle nitrogen stream at 45°C until dryness and reconstituted in 1 mL of mobile phase. The solution was filtered by syringe membrane (cellulose acetate, 0.2 *µ*m, Sigma, Singapore), collected in 2 mL glass vial, and subjected to UPLC-MS/MS analysis. The most important parameters in solid phase extraction are pH of samples. In this study, pH of the sample was changed from 2.0 to 5.0 with 0.5 increment steps for both SCX and MCX experiments and kept approximately at 6.5–7.0 for HLB experiments. For comparison, the best recovery was observed at pH 3 in case of mix-mode cation exchange column (MCX). The recovery of the seven compounds is depicted in Figure 5. As clearly shown in Figure 5, recoveries of seven target compounds ranged from 63% (PARA) to 110% (CARBA). The low recovery of PARA was also mentioned in previous studies [38, 39].

In conclusion, mix-mode cation exchange cartridge (MCX) was selected for enrichment and clean-up of seven pharmaceutical product residues in water samples. Therefore, the pH of the sample should be adjusted to 3.0 before treatment.

#### 3.2.4. Matrix Effect in UPLC-MS/MS

For addressing of ionization suppression/enhancement in UPLC-MS/MS, three sets of five water samples (surface water and hospital wastewater in a separated set) were prepared and spiked with a standard solution before and after SPE extraction. All samples were treated in the same manner as aforementioned. These samples were then subjected for UPLC-MS/MS measurement. The peak area of target analyte was used to calculate the matrix effect, extraction recovery, and overall recovery as instructed by Vu-Duc et al. [27]. The experimental results of matrix effect, extraction recovery, and overall recovery of all target compounds in both matrices are presented in Table 3.

As clearly demonstrated in Table 3, the overall recoveries of all target compounds are in the acceptable range (70% to 115%) in both surface water and hospital wastewater matrices, except for paracetamol (overall recovery 55 ± 12 and 56 ± 30, resp.). Ionization enhancement of KETO, CIPPRO, and OFLO was observed, whereas ion suppression phenomenon was observed in PARA, TRIM, and CARBA. In addition, there has been no effect observed for SULFA, and its recovery was good agreement in both surface water and hospital wastewater. However, the ionization enhancement/suppression of target compounds was in the acceptable range [26, 40, 41]. In addition, the entire recovery of all compounds was mainly contributed by the extraction step.

### 3.3. Environmental Application

The surface water and hospital wastewater (16 samples in total) were prepared using the aforementioned solid phase extraction procedure and were analyzed by UPLC-MS/MS at the optimum operating conditions. The concentration of all target compounds is shown in the influent and effluent of hospital wastewater (Table 4) and surface water (Table 5).

**Figure 6:**
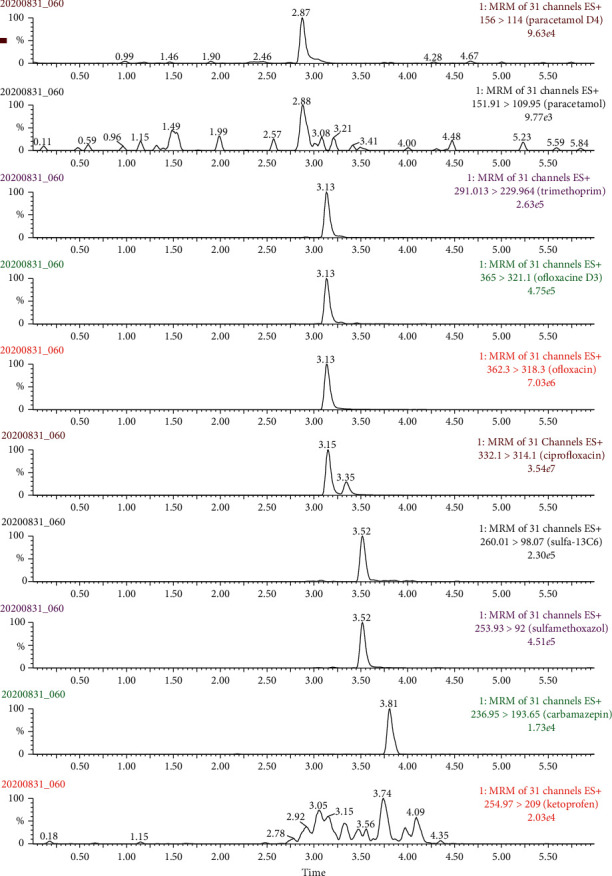
Extracted ion chromatograms of all target compounds in real hospital wastewater sample.

As can be seen in Table 4, TRIM and SULFA were detected in all influent samples; meanwhile, CARBA, CIPRO, PARA, and OFLO were only detected in some samples. In the influent wastewater, KETO and PARA were not found in all samples (Table 4). The highest concentration of SULFA (22.9 *µ*g L^−1^) and OFLO (18.0 *µ*g L^−1^) in the influent wastewater samples was observed. SULFA, PARA, and OFLO were also found at a high detection frequency in both influent and effluent of wastewater samples. However, concentration of all target compounds in effluent was found lower than that in the influent of wastewater samples. The extracted chromatogram of target compounds in hospital wastewater sample is demonstrated in Figure 6. The concentration of pharmaceutical products found in the hospital wastewater samples was also comparable with some recent studies [13, 22, 34]. For instantce, the concentration of SULFA in hospital wastewater has been found at a range from 0.31 *µ*g L^−1^ to 15.6 *µ*g L^−1^ according to the work of Tran et al. [13].

Only CARBA and SULFA were detected in surface water at an extremely low concentration (Table 5). The other compounds were below detection limit. This could be explained by the fact that all surface water samples were collected from West Lake, which is far from the hospital discharge. In addition, these surface water samples were collected in the summer (from April to September) in Vietnam. The sunlight and high activities of microorganisms could cause the decomposition for other compounds such as quinolones and ketoprofen. The finding of this study is also in good agreement with a recent publication [13].

## 4. Conclusion

In summary, the UPLC-MS/MS based analytical method has been optimized for the analysis of some pharmaceutical residues in water samples. All critical parameters of the method have been investigated. In the surface water of Hanoi, the most commonly found pharmaceutical product was sulfamethoxazole. This could be explained by the fact that recently sulfamethoxazole is the most popularly used pharmaceutical product in Hanoi. For further studies, UPLC-HRMS (time of flight MS/Orbitrap MS) will be used for finding the transformation products of pharmaceutical residues in water matrices. The degradation and removal of pharmaceutical product in environmental water using green absorbents will be investigated.

## Figures and Tables

**Table 1:** Operating conditions for target compounds in MS/MS mode in Waters Xevo-TQD mass spectrometer.

No.	Compounds	RT (min)	Ratio TQ/TC	Precursor ion	Product ions	Cone (V)	Collision energy (eV)
1	PARA	2.87	8.6	151.9	92.0	30	22
					109.9		18
2	PARA-D4	2.87	—	156.0	114	30	15
3	TRIM	3.13	1.6	291.0	229.9	28	24
					260.9		24
4	OFLO	3.14	2.5	362.3	261.3	25	20
					318.3		22
5	OFLO-D3	3.14	—	365.0	321.1	55	20
6	CIPRO	3.15	11.6	332.1	288.1	42	18
					314.1		22
7	SULFA	3.52	1.5	253.9	92.0	34	26
					156.9		16
8	SULFA–13C6	3.52	—	260.0	98.1	38	28
9	CARBA	3.81	1.2	236.9	178.9	42	34
					193.6		36
10	KETO	4.11	—	254.9	209.1	36	12

**Table 2:** Analytical figure of merits of seven major pharmaceutical residues by UPLC-MS/MS.

No.	Analytes	IS for quantification	Regression equation	*R* ^2^	MDL (*µ*g L^−1^)	
					Surface water	Hospital wastewater
1	PARA	PARA–D4	*y* = 0.0021 (±0.0001) x + 0.04 (±0.04)	0.9999	0.009	0.092
2	TRIM	PARA–D4	*y* = 0.0042 (±0.0001) x + 0.04 (±0.03)	0.9997	0.005	0.020
3	OFLO	OFLO–D3	*y* = 0.0020 (±0.0000) x − 0.02 (±0.01)	0.9990	0.010	0.016
4	CIPRO	OFLO–D3	*y* = 0.0021 (±0.0000) x − 0.01 (±0.01)	0.9931	0.007	0.123
5	SULFA	SULFA–13C6	*y* = 0.0010 (±0.0000) x − 0.10 (±0.00)	0.9998	0.006	0.024
6	CARBA	OFLO–D3	*y* = 0.0032 (±0.000) x − 0.0003 (±0.009)	0.9989	0.007	0.016
7	KETO	PARA–D4	*y* = 0.0026 (±0.0007) x + 0.03 (±0.18)	0.9988	0.015	0.014

**Table 3:** Matrix effect (ME), extraction recovery (RE), and overall recoveries (*R*) of target compounds.

No.	Analytes	ME ± SD (%)		RE ± SD (%)		*R* ± SD (%)	
		(*n* = 5)		(*n* = 5)		(*n* = 5)	
		Surface water	Wastewater	Surface water	Wastewater	Surface water	Wastewater
1	PARA	88 ± 8	81 ± 20	63 ± 19	69 ± 20	55 ± 12	56 ± 30
2	TRIM	83 ± 9	88 ± 12	91 ± 10	95 ± 15	75 ± 4	84 ± 18
3	OFLO	125 ± 20	115 ± 10	88 ± 15	80 ± 10	109 ± 19	92 ± 11
4	CIPRO	121 ± 40	101 ± 26	71 ± 8	72 ± 15	85 ± 18	73 ± 22
5	SULFA	99 ± 8	101 ± 6	90 ± 10	99 ± 7	89 ± 3	100 ± 4
6	CARBA	98 ± 8	90 ± 15	110 ± 14	102 ± 10	107 ± 11	92 ± 15
7	KETO	116 ± 8	120 ± 22	88 ± 8	96 ± 21	102 ± 12	115 ± 30

**Table 4:** Concentration of target pharmaceutical residues (*µ*g L^−1^) in hospital wastewater.

Analyte	In the influent					In the effluent					
	CT_IN	ND_IN	NHD1_IN	NHD2_IN	NHD3_IN	LS_EF	RHM_EF	CR1_EF	CR2_EF	ND_EF	NHD_EF
CARBA	1.75	0.10	<MDL	<MDL	<MDL	<MDL	<MDL	<MDL	<MDL	0.07	<MDL
OFLO	18.00	<MDL	<MDL	<MDL	<MDL	0.10	0.11	<MDL	0.06	0.06	<MDL
CIPRO	4.77	<MDL	<MDL	<MDL	<MDL	<MDL	<MDL	3.95	4.49	<MDL	<MDL
TRIM	0.33	1.91	0.40	0.07	0.12	0.35	<MDL	0.18	0.24	1.14	<MDL
SULFA	1.96	22.90	2.12	0.52	0.13	3.38	<MDL	1.58	1.91	0.10	<MDL
PARA	3.85	<MDL	<MDL	6.74	10.32	<MDL	<MDL	<MDL	<MDL	<MDL	<MDL
KETO	<MDL	<MDL	<MDL	<MDL	<MDL	<MDL	<MDL	<MDL	<MDL	<MDL	<MDL

**Table 5:** Concentration of target pharmaceutical residues (*µ*g L^−1^) in surface water.

Analyte	HT1	HT2	HT3	HT4	HT5
CARBA	0.032	<MQL	<MQL	<MQL	<MQL
OFLO	<MDL	<MDL	<MDL	<MDL	<MDL
CIPRO	<MDL	<MDL	<MDL	<MDL	<MDL
TRIM	<MDL	<MDL	<MDL	<MDL	<MDL
SULFA	0.081	0.027	<MQL	<MQL	<MQL
PARA	<MDL	<MDL	<MDL	<MDL	<MDL
KETO	<MDL	<MDL	<MDL	<MDL	<MDL

## Data Availability

The data used to support the findings of this study are included within the article.
